# Biomimetic Proteoglycans Strengthen the Pericellular Matrix of Normal and Osteoarthritic Human Cartilage

**DOI:** 10.1021/acsbiomaterials.4c00813

**Published:** 2024-08-12

**Authors:** Elizabeth R. Kahle, Hooman Fallahi, Annika R. Bergstrom, Anita Li, Colette E. Trouillot, Mary K. Mulcahey, X. Lucas Lu, Lin Han, Michele S. Marcolongo

**Affiliations:** † School of Biomedical Engineering, Science and Health Systems, Drexel University, Philadelphia, Pennsylvania 19104, United States; ‡ Department of Chemical and Biological Engineering, Villanova University, Villanova, Pennsylvania 19085, United States; § Department of Mechanical Engineering, Villanova University, Villanova, Pennsylvania 19085, United States; ∥ Department of Orthopaedic Surgery and Rehabilitation, Loyola University Medical Center, Maywood, Illinois 60153, United States; ⊥ Department of Mechanical Engineering, University of Delaware, Newark, Delaware 19716, United States

**Keywords:** biomimetic proteoglycan, pericellular matrix, osteoarthritis, IF-guided AFM, molecular engineering

## Abstract

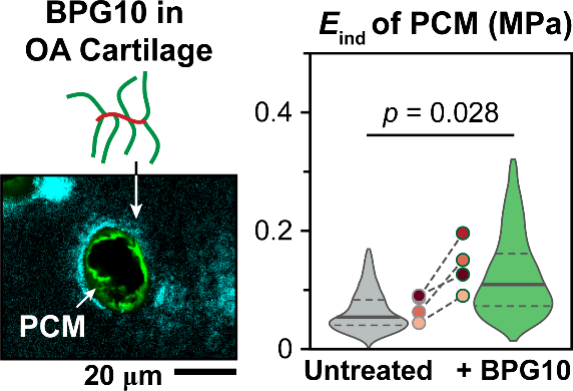

In osteoarthritis (OA), degradation of cartilage pericellular matrix (PCM), the proteoglycan-rich immediate cell microniche, is a leading event of disease initiation. This study demonstrated that biomimetic proteoglycans (BPGs) can diffuse into human cartilage from both normal and osteoarthritic donors and are preferentially localized within the PCM. Applying immunofluorescence (IF)-guided AFM nanomechanical mapping, we show that this localization of BPGs increases the PCM micromodulus of both normal and OA specimens. These results illustrate the capability of BPGs to integrate with degenerative tissues and support the translational potential of BPGs for treating human OA and other diseases associated with proteoglycan degradation.

## Introduction

In articular cartilage, chondrocytes reside in the pericellular matrix (PCM), a ≈ 2–4 μm thick intermediary microdomain that has distinct structure and composition from the bulk extracellular matrix (ECM).^1^ Given its immediate contact with cells, the PCM plays pivotal roles in mediating the biophysical and biological signals between chondrocytes and the ECM.^2^ In osteoarthritis (OA), degeneration of the PCM is one leading event of disease initiation, contributing to disrupted chondrocyte mechanotransduction, signaling, and eventually irreversible breakdown of cartilage.^3,4^ In the PCM, proteoglycans, and in particular, aggrecan, are preferentially localized therein.^5^ These proteoglycans are directly responsible for the mechanobiological functions of the PCM, and are often among the first molecular constituents to undergo degradation upon injury or disease initiation.^6^ Molecular engineering of the PCM integrity and its mechanobiological functions by targeting resident proteoglycans holds potential for modulating chondrocyte mechanotransduction and attenuating disease progression.^2^

We have synthesized a family of biomimetic proteoglycans (BPGs), which consist of chondroitin sulfate glycosaminoglycan (CS-GAG) side chains grafted onto a synthetic poly(acrylic acid) (PAA) core.^7−9^ These BPGs simulate the “bottle-brush” nanoarchitecture and the negatively charged nature of aggrecan. Recently, we showed that BPG10, a ∼ 180 kDa mimic with ∼7–8 CS-GAG bristles attached onto a ∼ 10 kDa PAA core, can passively diffuse through all zones of bovine cartilage explants *ex vivo* within 1 h.^10^ Also, following a single intra-articular injection into rabbit knee joints, BPG10 infiltrated throughout articular cartilage, and was retained in the matrix after 5 days.^11^ Following both *in vitro* and *in vivo* infiltrations, BPG10 was preferentially localized in the PCM and nearby territorial domain (T-ECM) through its molecular adhesion with native aggrecan.^12^ As a result, localization of BPG10 augmented the PCM micromodulus and promoted the intracellular calcium signaling of chondrocytes, demonstrating the potential of using BPG10 to modulate chondrocyte mechanobiology.^12^ Given its minimal toxicity, capability to rapidly diffuse throughout the full cartilage thickness, and retention within the cartilage matrix *in vivo*, BPG10 could serve as an intra-articularly injected OA therapeutic, which is minimally invasive compared to other commercial treatments such as microfracture, surgery and total or partial knee arthroplasty.^13^

Building on these findings, this study investigated the potential of BPG10 to diffuse into human cartilage specimens and determine its localization pattern within the matrix. We further evaluated the impact of BPG10 localization on the micromodulus of human cartilage PCM using immunofluorescence (IF)-guided, atomic force microscopy (AFM)-based nanomechanical mapping.^14^ Given that cartilage degradation in OA is hallmarked by the breakdown of PCM,^2^ we also tested if BPG10 can localize within a mildly degenerative PCM and impact its micromechanics in human cartilage specimens with signs of early OA. Together, results underscored the capability of BPG10 in strengthening the PCM in both normal and degenerative human cartilage, positioning BPG10 as a promising candidate for OA treatment in patients.

## Materials and Methods

### Synthesis and Functionalization of BPG10

Following our established procedures, BPG10 was synthesized by reaction of commercial CS-GAG (∼22 kDa, mixture of chondroitin-4-sulfate and chondroitin-6-sulfate GAGs, Sigma) in aqueous buffer and poly(acryloyl chloride) (PAC) (∼10 kDa, in 25% dioxane, PolySciences) in ethyl acetate (Fisher Scientific) at a 1:10 CS:PAC molar ratio.^9^ In brief, CS-GAGs (25 mg/mL) were dissolved in 0.1 M sodium borate buffer at pH 9.4. To this we added an equal volume of ethyl acetate containing the PAC solution (1:10 CS:PAC molar ratio). The reactants were stirred vigorously for 4 h on ice, followed by 4 h at room temperature. The product, contained in the water phase, was dialyzed against DI water for 4 days (water changes twice per day), then frozen and lyophilized. Fluorescently labeled BPG10 (DCCH-BPG10) was synthesized by periodate oxidation of the CS-GAG chains of BPG10 and subsequent conjugation with 7-diethylaminocoumarin-3-carboxylic acid, hydrazide (DCCH, Sigma).^9^

### BPG10 Diffusion Model and Confocal Microscopy Imaging

Normal human cartilage samples from deidentified donors were obtained from the National Disease Research Interchange (NDRI) and frozen in 1× PBS until use. Osteoarthritic human cartilage specimens were collected immediately after total knee replacement surgery from deidentified donors at the Hahnemann University Hospital, and were frozen in 1× PBS until use (Drexel University IRB #1503003490). For these OA samples, due to the large variability in sample size and shape resulting from surgical removal, roughly ∼4 mm biopsy punch plugs (*n* = 4, Collins grade 1) were used for all experiments. Additionally, 4 mm-wide cylindrical femoral cartilage plugs were harvested from *n* = 4 normal human cartilage specimens. Both normal and OA samples were incubated in 1× PBS with or without 10 mg/mL DCCH-BPG10 for 24 h in a dark room. Following the incubation, cartilage was embedded in optimal cutting temperature (OCT) media, and unfixed, 8 μm-thick sections were obtained via Kawamoto’s film-assisted cryo-sectioning.^15^ The sections were rinsed with 1× PBS to remove OCT and blocked with 10% goat serum (Life Technologies) for 20 min at room temperature. The sections were first fluorescently labeled with the primary antibody of collagen VI (70R-CR009X, Fitzgerald, 1:100 dilution), the PCM biomarker,^16^ for 20 min, rinsed twice with 1× PBS for 5 min each, and then, incubated with secondary antibody (goat anti-rabbit Alexa Fluor 488, AB150077, Abcam, 1:200 dilution) for 20 min in darkness. Sections were then rinsed twice for 5 min each with 1× PBS, and mounted with FluorSaveTM Reagent (345789, EMD Millipore). Confocal microscopy images were obtained using a Zeiss LSM 700 confocal microscope (Zeiss) at ex:405 nm for visualization of DCCH-BPG10 and ex:488 nm for collagen VI (repeated for all human samples). Internal negative controls were included following the same procedure but without the incubation with the primary antibody.

### Histology

Human cartilage samples were fixed in 4% paraformaldehyde for 24 h at 4 °C, dehydrated in graded ethanol and xylene, and then embedded in paraffin. Samples were sectioned into 6-μm-thick slices in the sagittal plane and stained with Safranin-O/Fast Green to assess the tissue morphology and gross-level staining of sulfated GAGs (sGAGs). The Mankin scoring metric^17^ was applied to grade each human sample by two blinded observers (HF and ARB). In addition, we assessed the loss of sGAGs by measuring the thickness of the surface layer that was devoid of sGAG staining (*t*_sGAG-devoid_) using ImageJ (≥60 measurements from ≥3 sections for each donor).

### Immunofluorescence-Guided AFM Nanomechanical Mapping

Cryo-sections of human cartilage specimens at ≈8 μm thickness in the sagittal plane were prepared in OCT media using Kawamoto’s film-assisted method.^15^ Immediately following fluorescent labeling of collagen VI, the PCM biomarker, the sections were tested using the Total Internal Reflection Fluorescence (TIRF)-AFM (MFP-3D, Asylum Research) in 1× PBS, following the established procedure.^3,18^ To delineate the micromodulus of PCM and T-ECM, within each 20 × 20 μm^2^ region of interest (ROI) with well-defined, ring-shaped PCM terrains, AFM nanomechanical mapping was performed in a 40 × 40 grid (1,600 indentations) using polystyrene microspherical tips (*R* ≈ 2.25 μm, nominal *k* ≈ 0.6 N/m, HQ:NSC36/tipless/Cr–Au, cantilever C, NanoAndMore) up to ≈120 nN maximum indentation force at 10 μm/s rate (≥3–5 ROIs for each sample). Also, to quantify the micromodulus of bulk interterritorial domain (IT-ECM), the nanomechanical mapping was performed in a 20 × 20 μm^2^ ROI with a 20 × 20 grid (400 indentations) in regions further removed from cells or PCM rings (≥3–4 ROIs for each sample). The effective indentation modulus, *E*_ind_, was calculated by fitting the entire loading portion of the indentation force-depth (*F-D*) curve to the finite thickness-corrected Hertz model.^19^ Using corresponding IF images of collagen VI, we separated the *E*_ind_ of PCM and T-ECM using a custom MATLAB (Mathworks) program, and excluded values corresponding to cell remnants.

### Statistical Analysis

Unpaired two-sample student’s *t*-test was applied to compare the *t*_sGAG-devoid_ between normal and early OA specimens, and nonparametric Mann–Whitney U test was applied to compare modified Mankin scores. Paired two-sample student’s *t*-test was applied to test the effect of BPG10 infiltration on the average values of *E*_ind_ from each donor for each of the PCM, T-ECM and IT-ECM domains. For all the statistical tests, the significance level was set at α = 0.05.

## Results

**Figure 1:**
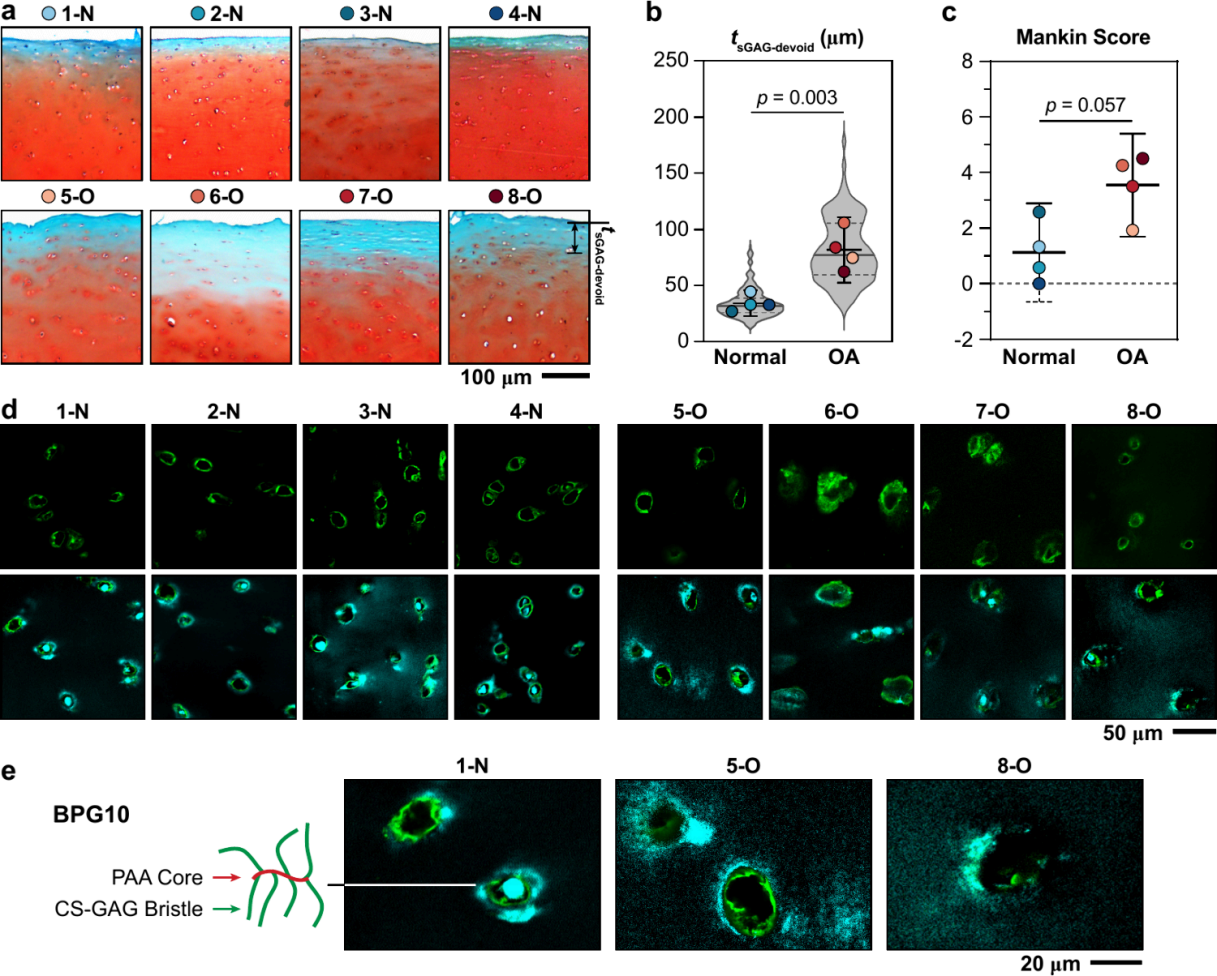
(a) Representative Safranin-O/Fast Green histology images of the eight human cartilage specimens, as well as the definition of superficial layer devoid of sGAGs, *t*_sGAG-devoid_. (b) Violin plot of *t*_sGAG-devoid_ for normal and osteoarthritic cartilage specimens. Each circle represents the average value measured from one donor (mean ±95% CI of the average values measured from each donor). (c) Modified Mankin scores for normal and osteoarthritic cartilage (mean ±95% CI). (d) Immunofluorescence (IF) images of human cartilage specimens infiltrated with fluorescently labeled DCCH-BPG10 (cyan) and costained with collagen VI (green) demonstrate the diffusion of BPG10 throughout cartilage matrix and its preferential distribution in the pericellular matrix (PCM) and the nearby territorial domain (T-ECM). (e) Left panel: Molecular architecture of BPG10, containing ∼7 CS-GAG side chains conjugated to the PAA backbone. Right panel: Representative zoomed-in IF images highlight the localization of BPG10 in the PCM and its expansion into the T-ECM.

We first analyzed the degree of cartilage degeneration for samples from the eight donors (Table 1). The four specimens with Collin’s grade 0 (1-N to 4-N) showed structural integrity representative of normal, undegraded cartilage.^20^ Specifically, these tissues displayed high intensity of Safranin-O staining, demonstrating the presence of proteoglycans and their sGAGs, smooth surfaces absent of fibrillation or fissures, as well as the absence of clustered chondrocytes arising from aberrant proliferation in OA (Figure 1a). For all four samples, there was an sGAG-absent superficial layer with an average thickness, *t*_sGAG-void_, of 34 ± 11 μm (mean ±95% CI, Figure 1b), which was typical for normal, adult human cartilage.^20^ In contrast, the four specimens with Collin’s grade 1 (5-O to 8-O) exhibited clear signs of degradation. These samples showed reduced overall sGAG staining, irregular surfaces with signs of fissures, as well as an increase in *t*_sGAG-void_ = 82 ± 29 μm (Figure 1a,b). As expected, these histological changes contributed to higher Mankin scores for the OA specimens with marginal significance (*p* = 0.057, Figure 1c). Despite these degenerative traits, we did not note increased cell clustering or appreciable cartilage erosion, supporting that these samples represented mild-to-moderate cartilage degeneration in early OA.

**Table 1:** Information about Cartilage Specimens Obtained from Normal and Osteoarthritic Donors

Collin’s grade	Donor no.	Designation	Mankin score	Sex of donor (male/female)	Age of donor (years)
0	1	1-N	1.33	F	61
	2	2-N	0.58	F	57
	3	3-N	2.58	F	61
	4	4-N	0.00	M	48
1	5	5-O	1.92	F	61
	6	6-O	4.25	F	68
	7	7-O	3.50	F	55
	8	8-O	4.50	M	59

For the OA specimens 5-O to 8-O, we found that collagen VI, the PCM biomarker,^16^ was localized within the pericellular domain of chondrocytes, similar to those of normal specimens 1-N to 4-N (Figure 1d). Therefore, the PCM of these OA tissues retained their compositional distinction from the bulk ECM. This was different from degenerated tissues that represent advanced OA, in which cartilage exhibits aberrant increased expression of PCM biomarkers such as collagen VI and perlecan, and their presence extends to the territorial matrix, leading to the loss of compositional distinction between PCM and the bulk T/IT-ECM.^20,21^ These specimens could thus serve as the model system for investigating the effects of BPG10 on the PCM in early degenerative stage. Following the incubation of cartilage samples with fluorescently labeled DCCH-BPG10, BPG10 was able to infiltrate into all eight specimens (Figure 1d). Furthermore, BPG10 was found to be preferentially localized in the PCM, with an expanded presence in the territorial domain (T-ECM) near the PCM, but a scarce presence in the bulk interterritorial domain (IT-ECM) (Figure 1d,e). This observation was consistent for both normal and OA cartilage samples, indicating that BPG10 retained its capability of preferentially distributing within the PCM of degenerative tissues.

**Figure 2:**
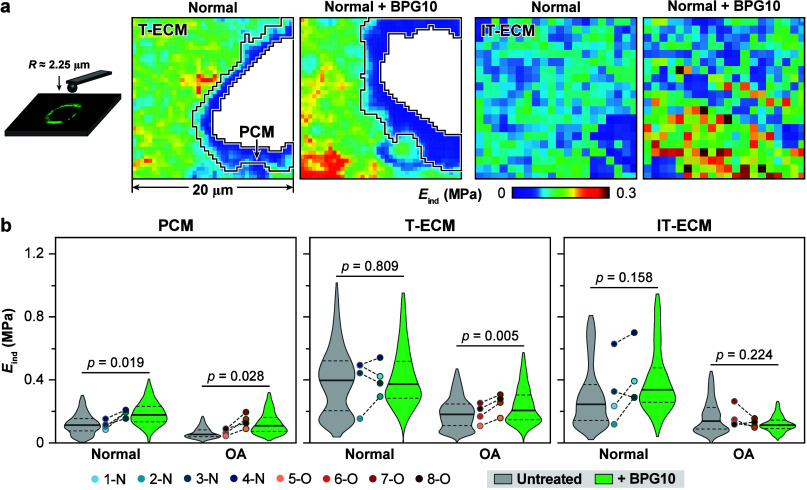
(a) Left panel: Schematic illustration of IF-guided AFM nanomechanical mapping on human cartilage cryosections using a microspherical tip (*R* ≈ 2.25 μm), where the PCM is immunolabeled with collagen VI. Right panel: Representative indentation modulus (*E*_ind_) maps of untreated and BPG10-treated normal human cartilage in 20 × 20 μm^2^ regions of interest (ROIs) either containing well-defined PCM rings (40 × 40 indents) or interterritorial domains (IT-ECM) further removed from cells (20 × 20 indents). Moduli corresponding to cell remnants were removed to increase clarity (white voids). (b) Violin plots of the PCM, T-ECM, and IT-ECM micromodulus for untreated and BPG10-treated cartilage (>470 locations for each region of each donor, *n* = 4 donors each for normal and OA groups). Each matched pair of circles represents the average modulus of untreated and BPG10-treated cartilage for the same donor from the same tissue region.

Applying IF-guided AFM nanomechanical mapping, we quantified the effects of BPG10 on cartilage micromechanics by comparing the micromodulus of PCM, T-ECM, and IT-ECM for untreated versus BPG10-infiltrated specimens (Figure 2a). For both normal and OA groups, infiltration of BPG10 led to marked stiffening of the PCM. The micromodulus of PCM increased by ≈72% from 114 ± 46 kPa to 188 ± 37 kPa for normal tissues, and by ≈102% from 71 ± 34 kPa to 140 ± 70 kPa for OA samples (mean ±95% CI of the average values from each donor, *p* < 0.05 for both groups, Figure 2b). For the nearby T-ECM domain, BPG10 also markedly increased the micromodulus of OA samples by ≈37%, but did not significantly impact the normal group. In contrast, for the IT-ECM, BPG10 did not show marked impacts on the micromechanics for either group. These results were in alignment with the preferred localization of BPG10 in the PCM and its expanded presence in the T-ECM (Figure 1d,e), evidencing the direct impact of BPG10 on augmenting the local matrix microenvironment. It is also worth noting that we found substantial variations of micromodulus across all eight specimens, both between and within each of the normal and OA group. For example, for untreated samples, the PCM modulus varied from 84 ± 5 kPa (1-N, mean ±95% CI from ≥470 indentation locations for each donor) to 152 ± 3 kPa (4-N) for the normal group, and from 44 ± 1 kPa (5-O) to 90 ± 2 kPa (8-O) for the OA group (Figure 2b). These substantial modulus variations were expected for human specimens, given the inherent large variability of human tissues due to differences in donor sex and age (Table 1), along with factors like historical joint use, disease history, and anatomical location.^22^ As a result of such variability, we only found marginal significance for the micromodulus of PCM (*p* = 0.051) and T-ECM (*p* = 0.053) between OA and normal groups, and no differences for the micromodulus of IT-ECM. Despite such variability, BPG10 consistently showed a marked strengthening effect of the PCM for both normal and OA specimens (Figure 2b).

## Discussion

Our results provide micromechanical evidence supporting BPG10 as a potential molecular therapeutic for OA intervention, as BPG10 can strengthen not only intact PCM in normal human cartilage, but also degenerative PCM in early OA specimens (Figure 2). This effect could be associated with its capability of interacting with aggrecan, the major proteoglycan in cartilage matrix.^23^ Our recent work suggests that when infiltrated into cartilage, BPG10 exhibits molecular adhesion with aggrecan through its “bottle-brush”-like, aggrecan-mimicking nanostructure.^12^ Also, the adhesion between BPG10 and aggrecan is at the same magnitude as aggrecan-aggrecan adhesion under physiological-like conditions.^12^ In cartilage matrix, aggrecan is more concentrated in the PCM,^5^ where it also undergoes more active turnover compared to the aggrecan residing in the bulk ECM.^24^ Therefore, these biophysical interactions enable BPG10 to be integrated with the aggrecan-rich PCM. Given that BPG10 contains negatively charged CS-GAG side chains (Figure 1e),^9^ its presence increases the fixed charge density of the PCM, contributing to the higher micromodulus observed in normal samples (Figure 2b). On the other hand, it is also possible that BPG10 could interact with other PCM-specific molecules, such as collagen VI, perlecan and biglycan,^2^ which could additionally contribute to its preferred localization in the PCM. These interactions will be investigated systematically in our future studies.

**Figure 3:**
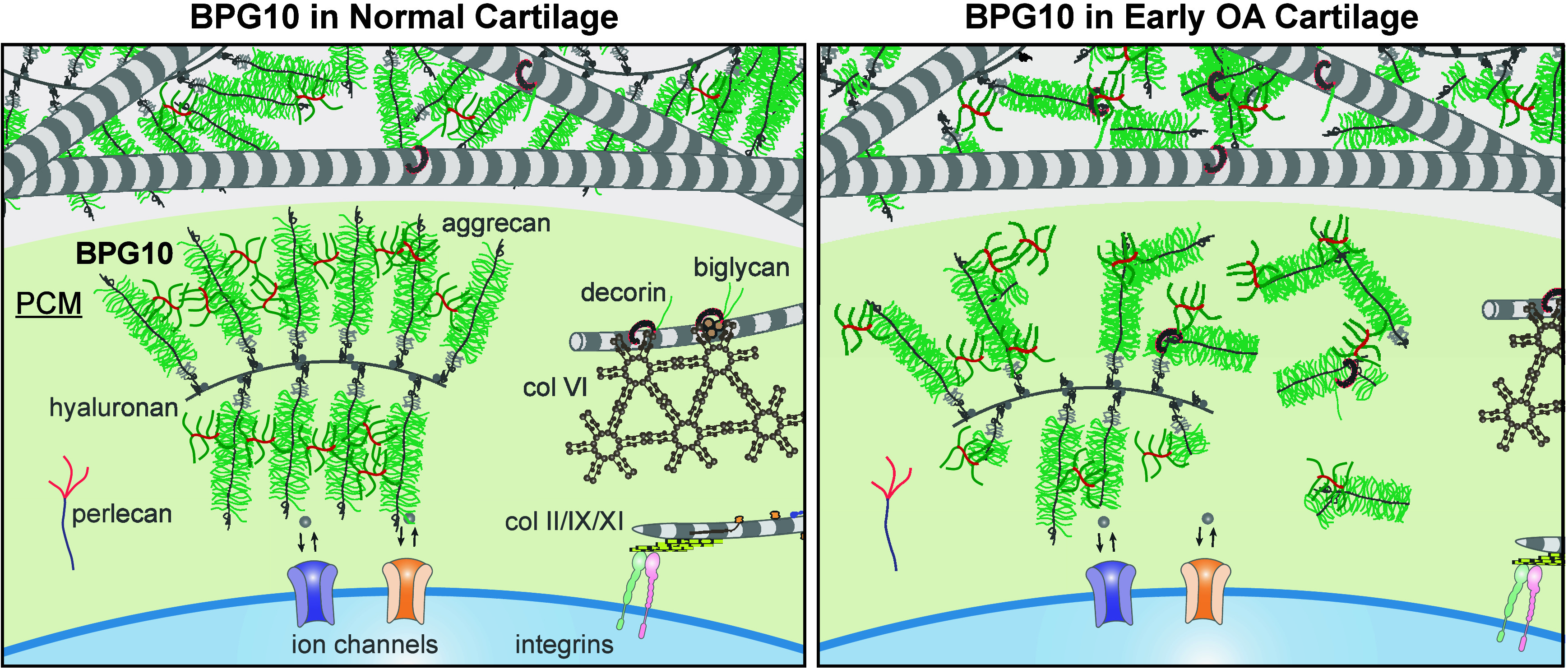
Schematic illustration of the working hypothesis of biomimetic proteoglycans in strengthening cartilage PCM. In the intact PCM of normal cartilage, BPG10 interacts with aggrecan-hyaluronan aggregates, which enables the integration of BPG10 with the native matrix, resulting in increased PCM micromodulus. In the degenerative PCM of early OA cartilage, BPG10 could interact with fragmented aggrecan. As a result, BPG10 could potentially offset the diffusive loss of fragmented aggrecan and fixed charges by its CS-GAG bristles, and in the meantime, increase the retention of aggrecan fragments. Therefore, BPG10 could potentially attenuate the degradation of PCM in early OA, and may rescue the disruption of downstream chondrocyte mechanotransduction by molecularly engineering the pericellular microniche.

In OA, aggravated chondrocyte catabolism results in elevated levels of aggrecanases and matrix metalloproteinases (MMPs), which cleave the aggrecan core protein at sites along its interglobular domain and CS-GAG domain.^25^ This results in aggrecan fragmentation and disassembly of aggrecan-hyaluronan aggregates, as the CS-GAG-containing aggrecan fragments lack the hyaluronan-binding, N-terminal G1 domain.^26^ This contributes to the diffusive loss of these unbound aggrecan fragments as OA progresses, leading to irreversible cartilage breakdown in advanced OA.^27^ It is worth noting that, in early OA, since the PCM is confined in the dense T/IT-ECM, these fragments remained localized within the PCM despite their dissociation from hyaluronan. In fact, likely due to elevated anabolism, there was even a temporary increase in the sGAG content in the degenerative PCM.^28^ Despite this increase in GAG content, the PCM still experienced a net reduction in micromodulus as the loss of aggrecan integrity likely outweighs the compensatory increase in its concentration.^3^ On the other hand, aggrecan fragments still partially retain their “bottle-brush”-like architecture and negatively charged CS-GAGs.^25^ Therefore, we postulate that the infiltration of BPG10 could also interact with aggrecan fragments to form interconnected supramolecular networks with the fragmented proteoglycans (Figure 3). By providing additional molecular adhesion, BPG10 could also increase the retention of aggrecan fragments, thereby delaying the breakdown of cartilage PCM. In addition, the presence of BPG10 in degenerative PCM could also contribute to a higher fixed charge density from its CS-GAG bristles, partially offsetting the effect of aggrecan fragment loss. This hypothesized role could explain the more profound impact of BPG10 on the T-ECM of OA cartilage, in which, we expect a higher proportion of aggrecan fragmentation relative to the normal group, and thus, a stronger effect of BPG10 in stabilizing fragmented aggrecan and augmenting the microenvironment. This structural effect of BPG10 is similar to our recently discovered role of decorin, a small leucine-rich proteoglycan, in providing physical linkages to increase the retention of aggrecan and its fragments in cartilage.^29^ This role of decorin is crucial for not only establishing proper cartilage biomechanical function and chondrocyte mechanobiology in normal cartilage,^29,30^ but also delaying cartilage breakdown in OA.^31,32^ To this end, by integrating with cartilage matrix, and especially the PCM, at the molecular level, BPG10 could recapitulate not only the biophysical properties of aggrecan by providing additional negative fixed charges, but also mimic the structural roles of regulatory small proteoglycans such as decorin to increase the retention and integrity of cartilage matrix.

Given its immediate contact with chondrocytes, degeneration of PCM and its residing proteoglycans is a leading event of OA, and precedes degradation of the bulk matrix and changes in tissue-level biomechanics.^3,4^ Since the PCM is pivotal in mediating chondrocyte mechanotransduction, degradation of PCM results in disrupted chondrocyte sensing, thereby contributing to the vicious loop of cartilage degradation.^2,3^ This renders the PCM a promising target for early OA intervention. Therefore, by augmenting the degenerative PCM, BPG10 could potentially rescue the disruption of chondrocyte mechanosensing. This hypothesis is supported by our recent work showing that infiltration of BPG10 into normal bovine cartilage promotes the *in situ* intracellular calcium signaling of chondrocytes,^12^ which is one of earliest cellular responses to mechanical stimuli^33^ and is positively correlated with chondrocyte anabolism.^34^ Furthermore, owing to it small molecular weight (≈ 180 kDa) and nanoscale hydrodynamic radius (≈ 60 nm),^9^ we have shown that infiltration of BPG10 throughout the full thickness of bovine cartilage was complete within 1 h,^10^ which was much faster than the intra-articular lymphatic clearance of small biopolymers (within 3–4 h).^35^ Also, its capability of retention in cartilage PCM for more than 5 days *in vivo* following one intra-articular injection^11^ indicates a potentially sustained effect of augmenting cartilage PCM and chondrocyte mechanosensing *in vivo*. To this end, one limitation of this study is that we cannot assess the chondrocyte mechanotransduction or metabolic changes of these frozen human cartilage specimens. Building on findings from this study, our ongoing work aims to test if BPG10 can restore normal chondrocyte mechanosensitive signaling in a degenerative environment, and if it can attenuate tissue-level cartilage sGAG loss and preserve biomechanical functions at the tissue level using live bovine and human cartilage explants, as well as *in vivo* animal models. We expect these results will establish the path for using BPG10 and other biomimetic proteoglycans to modulate chondrocyte mechanotransduction and slow OA progression. We will next test the dose-dependent effects of these biomimetic proteoglycans on chondrocyte mechanobiology to further enhance their efficacy for targeting OA intervention. Meanwhile, we note that the BPGs do not fully recapitulate the biophysical characteristics of native proteoglycans, such as the high molecular weight of full-length aggrecan (∼2.5 MDa) endowed by its high number (∼100) of CS-GAG bristles^36^ and the high collagen binding-affinity of decorin arising from its hydrophobic, leucine-rich protein core.^37^ Future modifications of BPGs to incorporate these molecular traits could further enhance their molecular engineering capabilities to achieve improved therapeutic outcomes.

## Conclusions

This study provides micromechanical evidence that biomimetic proteoglycans can strengthen the PCM of human cartilage for both normal and early OA specimens. We postulate that this function is associated with the capability of BPGs in interacting with native proteoglycans, such as aggrecan. As a result, BPGs have the potential to serve as a molecular therapeutic for modulating cell mechanotransduction and treating osteoarthritis. This biophysical role of BPGs may also be applicable to the intervention of other diseases associated with proteoglycan degradation, such as disc hernia, temporomandibular joint disorder, and cardiovascular diseases.

## References

[ref1] Heinegård D. Proteoglycans and more – from molecules to biology. Int. J. Exp. Pathol. 2009, 90, 575–586. 10.1111/j.1365-2613.2009.00695.x. 19958398 PMC2803248

[ref2] Guilak F.; Nims R. J.; Dicks A.; Wu C. L.; Meulenbelt I. Osteoarthritis as a disease of the cartilage pericellular matrix. Matrix Biol. 2018, 71–72, 40–50. 10.1016/j.matbio.2018.05.008. PMC6146061 29800616

[ref3] Chery D. R.; Han B.; Li Q.; Zhou Y.; Heo S. J.; Kwok B.; Chandrasekaran P.; Wang C.; Qin L.; Lu X. L.; Kong D.; Enomoto-Iwamoto M.; Mauck R. L.; Han L. Early changes in cartilage pericellular matrix micromechanobiology portend the onset of post-traumatic osteoarthritis. Acta Biomater. 2020, 111, 267–278. 10.1016/j.actbio.2020.05.005. 32428685 PMC7321882

[ref4] Wilusz R. E.; Zauscher S.; Guilak F. Micromechanical mapping of early osteoarthritic changes in the pericellular matrix of human articular cartilage. Osteoarthritis Cartilage 2013, 21, 1895–1903. 10.1016/j.joca.2013.08.026. 24025318 PMC3856176

[ref5] Poole A. R.; Pidoux I.; Reiner A.; Rosenberg L. An immunoelectron microscope study of the organization of proteoglycan monomer, link protein, and collagen in the matrix of articular cartilage. J. Cell Biol. 1982, 93, 921–937. 10.1083/jcb.93.3.921. 7119005 PMC2112142

[ref6] Lark M. W.; Bayne E. K.; Lohmander L. S. Aggrecan degradation in osteoarthritis and rheumatoid arthritis. Acta Orthop. Scand. Suppl. 1995, 66, 92–97. 10.3109/17453679509157660. 8553869

[ref7] Sarkar S.; Moorehead C.; Prudnikova K.; Schauer C. L.; Penn L. S.; Marcolongo M. Synthesis of macromolecular mimics of small leucine-rich proteoglycans with a poly(ethylene glycol) core and chondroitin sulphate bristles. Carbohydr. Polym. 2017, 166, 338–347. 10.1016/j.carbpol.2017.02.083. 28385241

[ref8] Prudnikova K.; Lightfoot Vidal S. E.; Sarkar S.; Yu T.; Yucha R. W.; Ganesh N.; Penn L. S.; Han L.; Schauer C. L.; Vresilovic E. J.; Marcolongo M. S. Aggrecan-like biomimetic proteoglycans (BPGs) composed of natural chondroitin sulfate bristles grafted onto a poly(acrylic acid) core for molecular engineering of the extracellular matrix. Acta Biomater. 2018, 75, 93–104. 10.1016/j.actbio.2018.05.013. 29753911

[ref9] Prudnikova K.; Yucha R. W.; Patel P.; Kriete A. S.; Han L.; Penn L. S.; Marcolongo M. S. Biomimetic proteoglycans mimic macromolecular architecture and water uptake of natural proteoglycans. Biomacromolecules 2017, 18, 1713–1723. 10.1021/acs.biomac.7b00032. 28398752

[ref10] Phillips E. R.; Haislup B. D.; Bertha N.; Lefchak M.; Sincavage J.; Prudnikova K.; Shallop B.; Mulcahey M. K.; Marcolongo M. S. Biomimetic proteoglycans diffuse throughout articular cartilage and localize within the pericellular matrix. J. Biomed. Mater. Res., Part A 2019, 107, 1977–1987. 10.1002/jbm.a.36710. 31056821

[ref11] Phillips E. R.; Prudnikova K.; Bui T.; Taylor A. J.; Galindo D. A.; Huneke R. B.; Hou J. S.; Mulcahey M. K.; Marcolongo M. S. Biomimetic proteoglycans can molecularly engineer early osteoarthritic cartilage in vivo. J. Orthop. Res. 2019, 37, 403–411. 10.1002/jor.24193. 30480335

[ref12] Kahle E. R.; Han B.; Chandrasekaran P.; Phillips E. R.; Mulcahey M. K.; Lu X. L.; Marcolongo M. S.; Han L. Molecular engineering of pericellular microniche via biomimetic proteoglycans modulates cell mechanobiology. ACS Nano 2022, 16, 1220–1230. 10.1021/acsnano.1c09015. 35015500 PMC9271520

[ref13] Jang S.; Lee K.; Ju J. H. Recent updates of diagnosis, pathophysiology, and treatment on osteoarthritis of the knee. Int. J. Mol. Sci. 2021, 22, 2619 10.3390/ijms22052619. 33807695 PMC7961389

[ref14] Kahle E. R.; Patel N.; Sreenivasappa H. B.; Marcolongo M. S.; Han L. Targeting cell-matrix interface mechanobiology by integrating AFM with fluorescence microscopy. Prog. Biophys. Mol. Biol. 2022, 176, 67–81. 10.1016/j.pbiomolbio.2022.08.005. 36055517 PMC9691605

[ref15] Kawamoto T.; Kawamoto K. Preparation of thin frozen sections from nonfixed and undecalcified hard tissues using Kawamot’s film method (2012). Methods Mol. Biol. 2014, 1130, 149–164. 10.1007/978-1-62703-989-5_11. 24482171

[ref16] Poole C. A.; Ayad S.; Schofield J. R. Chondrons from articular cartilage: I. Immunolocalization of type VI collagen in the pericellular capsule of isolated canine tibial chondrons. J. Cell Sci. 1988, 90, 635–643. 10.1242/jcs.90.4.635. 3075620

[ref17] Aigner T.; Cook J. L.; Gerwin N.; Glasson S. S.; Laverty S.; Little C. B.; McIlwraith W.; Kraus V. B. Histopathology atlas of animal model systems – overview of guiding principles. Osteoarthritis Cartilage 2010, 18, S2–S6. 10.1016/j.joca.2010.07.013. 20864020

[ref18] Wilusz R. E.; DeFrate L. E.; Guilak F. Immunofluorescence-guided atomic force microscopy to measure the micromechanical properties of the pericellular matrix of porcine articular cartilage. J. R. Soc. Interface 2012, 9, 2997–3007. 10.1098/rsif.2012.0314. 22675162 PMC3479909

[ref19] Dimitriadis E. K.; Horkay F.; Maresca J.; Kachar B.; Chadwick R. S. Determination of elastic moduli of thin layers of soft material using the atomic force microscope. Biophys. J. 2002, 82, 2798–2810. 10.1016/S0006-3495(02)75620-8. 11964265 PMC1302067

[ref20] Pritzker K. P. H.; Gay S.; Jimenez S. A.; Ostergaard K.; Pelletier J. P.; Revell P. A.; Salter D.; van den Berg W. B. Osteoarthritis cartilage histopathology: grading and staging. Osteoarthritis Cartilage 2006, 14, 13–29. 10.1016/j.joca.2005.07.014. 16242352

[ref21] Pullig O.; Weseloh G.; Swoboda B. Expression of type VI collagen in normal and osteoarthritic human cartilage. Osteoarthritis Cartilage 1999, 7, 191–202. 10.1053/joca.1998.0208. 10222218

[ref22] Treppo S.; Koepp H.; Quan E. C.; Cole A. A.; Kuettner K. E.; Grodzinsky A. J. Comparison of biomechanical and biochemical properties of cartilage from human knee and ankle. J. Orthop. Res. 2000, 18, 739–748. 10.1002/jor.1100180510. 11117295

[ref23] Han L.; Grodzinsky A. J.; Ortiz C. Nanomechanics of the cartilage extracellular matrix. Annu. Rev. Mater. Res. 2011, 41, 133–168. 10.1146/annurev-matsci-062910-100431. 22792042 PMC3392687

[ref24] Quinn T. M.; Dierickx P.; Grodzinsky A. J. Glycosaminoglycan network geometry may contribute to anisotropic hydraulic permeability in cartilage under compression. J. Biomech. 2001, 34, 1483–1490. 10.1016/S0021-9290(01)00103-8. 11672723

[ref25] Roughley P. J.; Mort J. S. The role of aggrecan in normal and osteoarthritic cartilage. J. Exp. Orthop. 2014, 1, 8 10.1186/s40634-014-0008-7. 26914753 PMC4648834

[ref26] Lohmander L. S.; Neame P. J.; Sandy J. D. The structure of aggrecan fragments in human synovial fluid. Evidence that aggrecanase mediates cartilage degradation in inflammatory joint disease, joint injury, and osteoarthritis. Arthritis Rheum. 1993, 36, 1214–1222. 10.1002/art.1780360906. 8216415

[ref27] Krishnan Y.; Grodzinsky A. J. Cartilage diseases. Matrix Biol. 2018, 71–72, 51–69. 10.1016/j.matbio.2018.05.005. PMC6146013 29803938

[ref28] Ojanen S. P.; Finnila M. A. J.; Reunamo A. E.; Ronkainen A. P.; Mikkonen S.; Herzog W.; Saarakkala S.; Korhonen R. K. Site-specific glycosaminoglycan content is better maintained in the pericellular matrix than the extracellular matrix in early post-traumatic osteoarthritis. PLoS One 2018, 13, e0196203 10.1371/journal.pone.0196203. 29694389 PMC5919041

[ref29] Han B.; Li Q.; Wang C.; Patel P.; Adams S. M.; Doyran B.; Nia H. T.; Oftadeh R.; Zhou S.; Li C. Y.; Liu X. S.; Lu X. L.; Enomoto-Iwamoto M.; Qin L.; Mauck R. L.; Iozzo R. V.; Birk D. E.; Han L. Decorin regulates the aggrecan network integrity and biomechanical functions of cartilage extracellular matrix. ACS Nano 2019, 13, 11320–11333. 10.1021/acsnano.9b04477. 31550133 PMC6892632

[ref30] Chery D. R.; Han B.; Zhou Y.; Wang C.; Adams S. M.; Chandrasekaran P.; Kwok B.; Heo S.-J.; Enomoto-Iwamoto M.; Lu X. L.; Kong D.; Iozzo R. V.; Birk D. E.; Mauck R. L.; Han L. Decorin regulates cartilage pericellular matrix micromechanobiology. Matrix Biol. 2021, 96, 1–17. 10.1016/j.matbio.2020.11.002. 33246102 PMC7902451

[ref31] Li Q.; Han B.; Wang C.; Tong W.; Wei Y.; Tseng W.-J.; Han L.-H.; Liu X. S.; Enomoto-Iwamoto M.; Mauck R. L.; et al. Mediation of cartilage matrix degeneration and fibrillation by decorin in post-traumatic osteoarthritis. Arthritis Rheumatol. 2020, 72, 1266–1277. 10.1002/art.41254. 32162789 PMC7486252

[ref32] Han B.; Li Q.; Wang C.; Chandrasekaran P.; Zhou Y.; Qin L.; Liu X. S.; Enomoto-Iwamoto M.; Kong D.; Iozzo R. V.; Birk D. E.; Han L. Differentiated activities of decorin and biglycan in the progression of post-traumatic osteoarthritis. Osteoarthritis Cartilage 2021, 29, 1181–1192. 10.1016/j.joca.2021.03.019. 33915295 PMC8319061

[ref33] Clapham D. E. Calcium signaling. Cell 2007, 131, 1047–1058. 10.1016/j.cell.2007.11.028. 18083096

[ref34] Weber J. F.; Waldman S. D. Calcium signaling as a novel method to optimize the biosynthetic response of chondrocytes to dynamic mechanical loading. Biomech. Model. Mechanobiol. 2014, 13, 1387–1397. 10.1007/s10237-014-0580-x. 24696123

[ref35] Doan T. N.; Bernard F. C.; McKinney J. M.; Dixon J. B.; Willett N. J. Endothelin-1 inhibits size dependent lymphatic clearance of PEG-based conjugates after intra-articular injection into the rat knee. Acta Biomater. 2019, 93, 270–281. 10.1016/j.actbio.2019.04.025. 30986528 PMC7523704

[ref36] Lee H.-Y.; Han L.; Roughley P. J.; Grodzinsky A. J.; Ortiz C. Age-related nanostructural and nanomechanical changes of individual human cartilage aggrecan monomers and their glycosaminoglycan side chains. J. Struct. Biol. 2013, 181, 264–273. 10.1016/j.jsb.2012.12.008. 23270863 PMC3578138

[ref37] Gubbiotti M. A.; Vallet S. D.; Ricard-Blum S.; Iozzo R. V. Decorin interacting network: A comprehensive analysis of decorin-binding partners and their versatile functions. Matrix Biol. 2016, 55, 7–21. 10.1016/j.matbio.2016.09.009. 27693454 PMC6938589

